# Some Diseases of the Hock-joint1Read before the Illinois State Veterinary Medical Association, November, 1895.

**Published:** 1896-02

**Authors:** W. J. Martin

**Affiliations:** Kankakee, Ill.


### SOME DISEASES OF THE HOCK-JOINT.[1]

BY W. J. MARTIN, V.S.,
KANKAKEE, ILL.

THE hock-joint is one of the most important articulations of the equine body, and none is subjected to so many pathological evils as it. This joint has engaged the attention of the veteriinary pathologist from remote times, and, from the number of cases of lameness which we meet with in practice, would seem to bear out the correctness of an assertion made to me many years ago by an eminent veterinary practitioner, that 90 per cent. of lameness in the posterior extremities was due to diseased conditions of the hock-joint.

Viewed from the standpoint of a naturalist, the equine tarsus presents many points of interest. The first of these is its complex structure, which is the result of a long period of metamorphosis, or change, in which nature has endeavored to adapt itself to the animal's changed surroundings, due to domestication. We see in those extinct fossil equidæ, which preceded our existing horse family in geological times, hock-joints of very simple construction. For instance, in the family Hypotherium, the nearest extinct relative of the present horse family, the joints

[1] Read before the Illinois State Veterinary Medical Association, November, 1895.

are of very simple construction. The small bones in the hock of hypotherium are articulated directly with each other and with the long bones, whereas in our modern horse the small bones interlock each other, and also the long bones, thus forming at the same time a stronger and more complicated joint, one which is well adapted to resist the strain imposed upon it by domestication, but at the same time one which is much more predisposed to the ravages of disease.

The hock-joint presents for our consideration two well-marked mechanical movements, viz.: A true hinge-movement and a gliding movement. The hinge-movement is found in the tibia articulating with the grooved surface of the artragalus. The gliding movement is confined to the cuneiform magnum, medium, parsum, and os cuboid.

The diseases of the hock-joint have been grouped into two grand divisions, viz.: diseases of the osseous structures and diseases of the soft parts, such as the skin, muscles, fascia, ligaments, etc.

Owing to the scope of the subject, which a minute study of the diseases of the hock-joint involves, it is not possible in one short essay to enter fully into a complete description of all the diseases of this joint. I will, therefore, confine myself to a consideration of some of the diseases of the soft parts.

Capped Hock. Beginning at the summit of the calcaneum we find a diseased condition, termed hygroma or capped hock. There are two forms of this malady. The most important one is due to an enlargement of the synovial bursæ, between the gastrocnemius internus and externus muscles This lesion in some cases gives rise to much pain and lameness. It is usually caused by a sprain or injury from forced efforts at heavy draft or in jumping. The second, or serous, is comparatively simple in nature, and is generally caused by a bruise, such as kicking in the stall or in lying down on a bare plank floor. Some writers mention caries or necrosis of the summit of the calcaneum, though I must confess that this must be of extremely rare occurrence. Other conditions which give rise to enlargement of the joint of the hock are dropsical effusions due to lymphangitis or debility due to wasting diseases.

*Treatment.* In those cases caused by an injury of any kind, and where there is much heat and pain, and the animal is in full condition, I generally give a mild alœtic purge, followed by salines in the drinking water, such as Epsom salts or nitrate of potash. I also find great benefit in allowing the animal the freedom of a small yard or box stall. The therapeutic agents to be applied to the hock are of the cooling and discutient order. If in summer pounded ice placed in a sack and adjusted over the os calcis answers admirably. If ice cannot be had cold water may be used, followed by smart hand friction with strong camphor spirits. In the course of a week or ten days after the inflammation has subsided and the effusion still remains, the treatment then to be pursued is one of absorption. For this purpose I find nothing better than the glycerin of iodine solution, made thus:

℞.—Iodine et Iodide Potassium . . . āā ℥j

Glycerin Puræ . . . . . . ℥ij

Fiat a solution by means of a water-bath and paint the enlargement once or twice a day with the mixture.

This remedy in my hands has succeeded the actual cautery, seton, puncturing with the aspirator, etc. In treating capped hock with this mixture it possesses the additional advantage of leaving no scar or blemish and can be used while the horse is at his work. I am decidedly opposed to the use of the hot iron or seton in treating capped hock, for in many cases we find the areolar tissue and other soft parts indurated and permanently calloused by their use, thereby leaving an unsightly blemish, which would not have been the case if a patient system of absorption had been pursued. Some practitioners inform me that they derive great benefit from the use of a thick pad adjusted over the enlargement, so as to prevent the animal from bruising it in lying down at night.

CURB. From the seat of capped hock we pass downward on the posterior part of the hock and meet with that pathological condition known as "curb."

*Definition.* Curb is a sprain of the calcaneum cuboid ligament. That the above definition is somewhat dogmatic we must admit, because there is a wide difference of opinion among veterinarians the world over concerning the true seat of this disease. Youatt, Spooner, and many others of the old veterinary authors claimed that it was caused by a sprain of the calcaneum cuboid ligament, while Percivall claimed that it was caused by a sprain or rupture of the synovial sheath between the post annular ligament and the tendon of the perforatus muscle. Prof. Williams says that it is caused by a sprain of the calcaneum cuboid ligament, as do likewise Profs. Liautard, Smith, and others. On the other hand, Prof. Pritchard, of the Royal Veterinary College of London, who, we must admit, is a very good authority, says that curb is due to an inflammation of the synovial sheath of the posterior annular ligament and the tendon of the flexor perforatus muscle. In a lecture on curb, delivered before the Lincolnshire Veterinary Medical Association, referring to Prof. Williams's assertion that curb is a sprain of the calcaneum cuboid ligament, he says: "You can call to mind the strength and position of the calcaneum cuboid ligament; it is one of the most powerful ligaments in the body. I have never yet seen it in a diseased condition, except through an injury, such as a kick from a horse. I have never yet seen the slightest trace of disease of this ligament, and if you examine the strength of it you will come to the conclusion that it would be an enormous effort on the part of an animal that could possibly injure it. It runs down the back of the os calcis. It is half an inch in thickness, and is firmly implanted in the cuboid bone, and is strong enough to hang a horse on."

We must admit the position taken by Prof. Pritchard is a strong one, but, in my humble opinion, based upon numerous dissections and years of close observation, his theory is not correct in many cases. I have found well-marked disease of the calcaneum cuboid ligament in curb, and in other cases disease of the synovial membranes, and even of the tendons and os calcis itself, so that it is just as well not to be too dogmatic in regard to the pathology of this disease. In regard to the soundness or unsoundness of a horse having a curb, when the horse is of mature years and the formation of the hock is good, I would have no hesitation in saying that practically such a horse was sound, though technically such an animal could not be considered so. A horse with a bent or so-called "sickle hock" should be viewed with much suspicion, and I do not hesitate to say that such animals are not to be trusted for hard work.

In the matter of curb being an hereditary evil of the horse family, my belief is that a stallion or mare with curby hocks is most likely to transmit the same defective anatomical conformation to their offspring.

*Treatment.* In severe cases of curb where there is much lameness, together with heat and pain, it is well to allow the animal perfect repose, and use cooling and refrigerant lotions to the hock. After a few days these may be discontinued and strong camphor spirits may be thoroughly applied with strong hand-rubbing. When the heat and pain have subsided it is well to remove the ordinary shoe and substitute one that is very thin at the toe and gradually becomes thicker backward until at the heels a thickness of an inch and a half has been attained. This relieves the tension on the tendon and ligaments, and at the same time serves to prevent a return of the malady.

As to removing the exudation which may remain, any of the resolvent remedies may be used. Of these I prefer the glycerin of iodine perseveringly applied. In cases of curbs of long standing, in which the parts are much enlarged and indurated, rendering the animal quite lame, I have also derived beneficial results from the compound solution of mercury, made thus:

| | |
|---|---|
| ℞.—Hydrargyri Iodidum Rubrum . . . | ʒiv |
| Iodide Potassium . . . . . . | ʒiv |
| Sp. Vini Rect. . . . . . . . | ℥iv |

Sig.—To be applied to the enlargement once a day if required.

In some obstinate cases which refused to yield to the above treatment, I have used the pointed iron with good results.

